# Targeting Wnt Signaling for the Treatment of Gastric Cancer

**DOI:** 10.3390/ijms21113927

**Published:** 2020-05-30

**Authors:** Sarah Koushyar, Arfon G. Powell, Elizabeth Vincan, Toby J. Phesse

**Affiliations:** 1 European Cancer Stem Cell Research Institute, Cardiff University, Cardiff CF24 4HQ, UK; koushyars@cardiff.ac.uk (S.K.); powellA16@cardiff.ac.uk (A.G.P.); 2 Division of Cancer & Genetics, Cardiff University, Cardiff CF14 4XW, UK; 3 Victorian Infectious Diseases Reference Laboratory, Doherty Institute for Infection and Immunity, University of Melbourne, Melbourne, VIC 3000, Australia; e.vincan@unimelb.edu.au; 4 School of Pharmacy and Biomedical Sciences, Curtin University, Perth WA 6102, Australia; 5 The Peter Doherty Institute for Infection and Immunity, University of Melbourne, Melbourne VIC 3000, Australia

**Keywords:** Wnt, Fzd, Fzd7, stomach, gastric, cancer, signaling

## Abstract

The Wnt signaling pathway is evolutionarily conserved, regulating both embryonic development and maintaining adult tissue homeostasis. Wnt signaling controls several fundamental cell functions, including proliferation, differentiation, migration, and stemness. It therefore plays an important role in the epithelial homeostasis and regeneration of the gastrointestinal tract. Often, both hypo- or hyper-activation of the pathway due to genetic, epigenetic, or receptor/ligand alterations are seen in many solid cancers, such as breast, colorectal, gastric, and prostate. Gastric cancer (GC) is the fourth commonest cause of cancer worldwide and is the second leading cause of cancer-related death annually. Although the number of new diagnoses has declined over recent decades, prognosis remains poor, with only 15% surviving to five years. Geographical differences in clinicopathological features are also apparent, with epidemiological and genetic studies revealing GC to be a highly heterogeneous disease with phenotypic diversity as a result of etiological factors. The molecular heterogeneity associated with GC dictates that a single ‘one size fits all’ approach to management is unlikely to be successful. Wnt pathway dysregulation has been observed in approximately 50% of GC tumors and may offer a novel therapeutic target for patients who would otherwise have a poor outcome. This mini review will highlight some recent discoveries involving Wnt signaling in GC.

## 1. Gastric Cancer 

Gastric cancer (GC) is the sixth commonest cause of cancer worldwide and the second leading cause of cancer-related death [1], and five-year survival rates remain at approximately 18% [2]. Treatment options are tailored to each individual patient’s needs but are largely stratified as palliative or curative. Following diagnosis, patients undergo a series of investigations to determine a clinical stage based on the tumor node metastasis (TNM) staging system [3]. These investigations consist of computer tomography (CT) scanning of the thorax, abdomen, and pelvis; staging laparoscopy; and in selected cases, positron emission tomography (PET) CT scanning. Patients with evidence of distant metastasis or those who are based on concurrent illnesses are commonly offered palliative treatment [4]. 

GC can be histologically characterized using the Lauren classification, with tumors described as either gland-forming adenocarcinoma (intestinal type) or poorly differentiated with infiltrative single cell morphology (diffuse type) [5]. These two subtypes are distinct entities that differ in their pathogenesis, etiology, and epidemiology; however, a small number of gastric adenocarcinomas can present with features of both subtypes [6]. The intestinal subtype is more commonly diagnosed in high-risk populations, such as older males, and tumorigenesis is often associated with *H. pylori* infection [6]. A variation of the diffuse type is the signet ring cell adenocarcinoma and is associated with a poor prognosis [7]. Depending on both the degree of local tumor invasion and location of the primary tumor, surgical resection with total or subtotal gastrectomy and radical lymphadenectomy remains the only treatment modality [8]. The Southwest Oncology Group (SWOG 9008) trial revealed that adjuvant chemoradiation in patients undergoing potentially curative surgery for GC or junctional esophageal cancer (OC) was associated with improved overall survival (OS); however, the post-operative chemoradiation was poorly tolerated [9]. Furthermore, the Medical Research Council Adjuvant Gastric Infusional Chemotherapy (MAGIC) trial showed that perioperative chemotherapy (Epirubicin, Cisplatin, and 5- Fluorouracil) in patients with GC or junctional OC had a significantly higher OS and progression-free survival (PFS) when compared to patients who had surgery alone [10]. Unfortunately, there are a number of patients who develop chemotherapy-related morbidity, and therefore, simply offering chemotherapy to all patients is not a viable treatment option. 

The prognosis for patients with metastatic GC is very poor, with a median survival varying from 4 to 9 months depending on the degree of metastatic disease and whether patients receive palliative chemotherapy [11]. Although there is a greater understanding of the etiology and pathophysiology of GC, identifying novel and reliable therapeutic targets is a challenge facing academics and clinicians alike. There is growing evidence highlighting the central role of the Wnt signaling pathway in GC development and progression. This review will explore the Wnt signaling pathway in both the initiation and progression of GC, and how the pathway can be therapeutically targeted. 

## 2. Wnt Signaling

There are 19 highly conserved Wnt ligands, described as secreted morphogens that carry out their function from medium to long distance ranges that elicit several signaling pathways. Whether Wnt ligands act locally or distantly is dependent on how the Wnt ligands are released. Wnt ligands can be released from the plasma membrane directly, as part of an exosome or lipid protein particles, or can be tethered to the plasma membrane [12]. The varying mechanisms of how Wnt ligands are released explains their diverse role during the development and maintenance of organs. Wnt ligands can bind, with varying affinities, to a heterodimeric receptor complex of Frizzled receptors (Fzd1-10) and their co-receptors, low-density lipoprotein receptor-related protein 5/6 (LRP5/6), receptor tyrosine kinase-like orphan receptor 2 (ROR2), and related to receptor tyrosine kinase (Ryk), to initiate either β-catenin-dependent (canonical) or β-catenin-independent signaling (non-canonical) [13]. Wnt proteins are palmitoylated by the o-acyl transferase porcupine [14], which, together with Wntless/Evi, is required for the secretion of Wnt ligands [12,15]. Members of the R-spondin (Rspo) family are also extracellular positive regulators of Wnt signaling. Rspo binds to leucine-rich repeat containing G protein-coupled receptors 4-6 (LGR4-6), preventing the activity of the two homologues E3 ubiquitin ligases RNF43 and ZNRF3, leading to an accumulation of Fzd receptors on the cell surface. However, in the absence of Rspo binding, Fzd receptors are targeted for lysosomal degradation by RNF43/ZNRF3 [12]. 

In the absence of Wnt signaling, cytoplasmic β-catenin is targeted by ubiquitin-directed degradation by the intracellular regulator: The β-catenin destruction complex [15]. This destruction complex is composed of the intracellular scaffold proteins AXIN, adenomatous polyposis coli (APC), casein kinase 1α (CK1α), and glycogen synthase 3 (GSK3α/β), which targets β-catenin by catalyzing the phosphorylation of a phospho-degron at the N-terminus. Activation of the canonical Wnt pathway, through the binding of canonical Wnt ligands (such as Wnt3a), causes dimerization of the Fzd receptor and LRP5/6 co-receptor, and leads to phosphorylation of the cytoplasmic tail of LRP5/6, recruiting disheveled (DVL) and AXIN to the cell membrane [15]. The destruction complex is now no longer able to degrade β-catenin, leading to an increase of cytoplasmic β-catenin. β-catenin then translocates to the nucleus, where it acts as a transcription co-activator, by binding to members of the T-cell factor/lymphoid enhancer factor (TCF/LEF) family, and CREB-binding protein (CBP) to modulate transcription of Wnt target genes. In the absence of canonical Wnt signaling, TCF/LEF family activity is repressed by interaction of the Groucho/ transducin-like enhancer of split (TLE) family of co-repressors [15]. The canonical Wnt signaling pathway is essential for determining the cell fate and the proliferation and self-renewal of both stem and progenitor cells [16]. Recently, two transcriptional regulators of the Hippo pathway, YAP/TAZ, have been identified as novel regulators of Wnt signaling [12]. In the absence of Wnt ligands, YAP/TAZ form part of the destruction complex, recruiting β-TrCP, and dampening Wnt signaling. YAP/TAZ have a dual role in Wnt signaling, as activation of the signaling cascade causes displacement of YAP/TAZ and β-TrCP from AXIN1 by LRP during degradation of the destruction complex. The contradicting role of YAP/TAZ in Wnt signaling highlights that there is substantial crosstalk between the Wnt and Hippo pathway [12]. 

The non-canonical Wnt signaling pathway occurs via a transcriptional β-catenin-independent signal transduction and can be broadly categorized into the planar cell polarity (PCP) and the Wnt/calcium (Wnt/Ca^2+^) pathway [17]. Binding of Wnt5a, Wnt7a, Wnt8a, or Wnt11 (non-canonical Wnts) to Fzd receptors and their respective co-receptors ROR2 and RYK initiates the PCP pathway. The PCP pathway was first described in *Drosophila* and is determined by the asymmetrical location of core PCP components (proximal and distal subsets). The proximal subset includes the atypical cadherin flamingo (FMI), the LIM domain protein prickle (PK), and the Van Gogh transmembrane protein (VANG1/2). The distal subset includes Fmi, Fzd receptors, DVL, and ankyrin repeat protein Diego (DGO), and it is the intercellular interactions between these subsets that allow for the asymmetrical localization of these core PCP proteins [16]. 

Rac1 and RhoA (Rho GTPases) are effectors of cytoskeletal rearrangement. Activation of PCP signaling causes both RhoA and Daam1 to interact with DVL, leading to ROCK1 activation, mediating cytoskeletal rearrangement [16]. The disparity in Wnt ligand/receptor complexes allows for variation in the transmission of signaling, resulting in the activation of genes, such as *c-jun*, *Cdc42*, and *DVL*. PCP can also be activated upon binding of syndecan4 (SDC4) and R-SPO to Fzd7, transmitting Wnt5a (non-canonical Wnt)-induced internalization of the ligand/receptor complex [17]. 

In vertebrates, Wnt ligands can activate Ca^2+-^dependent events through the release of intracellular Ca^2+^. The Wnt/Ca^2+^ was first identified in zebrafish, where overexpression of Wnt5a induced an increase in Ca^2+^ signaling, and in *Xenopus* caused the activation of protein kinase C (PKC) [18,19]. Initiation of Wnt/Ca^2+^ signaling through Fzd/ROR1/2 interaction activates inositol 1,4,5-triphosphate (IP3), 1,2-diacylglycerol (DAG) and Ca^2+^ by phospholipase C (PLC). PLC is responsible for the modification of IP3 and DAG, allowing IP3 to interact with Ca^2+^ channels on the endoplasmic reticulum, thus causing the release of Ca^2+^ ions. DAG can then work with Ca^2+^ ions to activate PKC. Further, Ca^2+^ activates calmodulin-dependent protein kinase II (CaMKII), which works with PKC to activate both CREB and NFκB to regulate gene transcription [19]. 

Recently, a novel arm of non-canonical Wnt signaling known as the Wnt/STOP pathway (Wnt-dependent stabilization of proteins) was discovered. Canonical Wnt signaling peaks during the G2/M phase of the cell cycle as LRP6 is primed by Cyclin Y/CDK14, and at this point, activation of β-catenin-independent stabilization of proteins is seen, leading to inhibition of GSK-3β activity. This prevents degradation of multiple proteins, resulting in an increased cell protein content, essential for cellular division [16].

We do not fully understand how Wnt ligands travel between cells to exert their long-range signaling activity, with both secreted- and membrane-associated mechanisms observed. Extracellular factors, such as *Swim* [20], *Frzb*, and *Cres* [21], can promote the solubility and diffusion of Wnt proteins and enhance their activity. However, membrane-associated mechanisms have also been identified, including the transport of Wnt in exosomes [22] and cytonemes (dynamic actin-based membrane structures known as signaling filopodia) [23]. Interestingly, cytoneme formation is regulated by Wnt signaling to activate paracrine Wnt/PCP signaling, whilst in neighboring cells, cytoneme-associated Wnt8a activates β-catenin-dependent Wnt signaling [24].

## 3. Deregulated Wnt Components in GC

Genomic analysis identified 46% (range 43%–48%) of gastric tumors exhibit deregulation of the Wnt/β-catenin pathway [25]. Several Wnt ligands are upregulated in human gastric tumors, including *WNT1* [26], *WNT2b* [27], *WNT5a* [28], *WNT6* [29], and *WNT10a* [30] (Table 1). These Wnt ligands are also upregulated in the gastric tumors of gp130*^F/F^* mice [31]. 

Moreover, in 13/15 GC cell lines, nuclear localization of endogenous β-catenin is observed, with a subsequent increase in TCF/LEF transcriptional activity, confirming aberrant canonical Wnt signaling in GC cells [32]. Loss of function of APC is often due to hypermethylation of the promoter region or gene mutation. Wang et al. [33] found 9 out of 16 high-grade gastric adenomas had methylation of the *APC* promoter, which correlated with the grade of dysplasia and abnormal expression of β-catenin. Deregulation of Wnt signaling by truncation of *Apc* is able to trigger tumorigenesis in the antrum [31] or the corpus [34] in mice, although compound mutant *Kras*:*Apc* mice are more commonly used in experiments to investigate the cell of origin in the corpus [35]. Further, the Kyoto Encyclopedia of Genes and Genomes (KEGG) pathway database identifies the Wnt pathway as the third most active in gastric tumors, with *RNF43*, *AXIN1/2*, *CTNNB1*, and *APC* frequently mutated [5]. Additionally, almost 30% of microsatellite instability (MSI) high GCs have a frameshift mutation in *AXIN2* [36] and genetic deletion of *Gsk3β*, leading to aberrant Wnt signaling, which drives rapid gastric tumor formation in mice [37]. 

The non-canonical Wnt pathway has also been implicated in GC. High protein expression of the non-canonical Wnt5a ligand was observed in 71 out of 237 primary GC patient samples (both intestinal and diffuse type), and these samples were positively associated with the depth of invasion and the degree of lymph node metastasis [38]. Further, an in vivo xenograft model showed that the injection of metastatic GC cells with stable Wnt5a knockdown into the spleen of nude mice significantly decreased the number of liver metastatic nodules when compared to control GC cell lines [39]. Wnt5a also regulates GC migration and invasion in vitro and treatment with an anti-Wnt5a antibody significantly reduced the number of metastatic liver foci compared to a control antibody in xenografts [40]. However, this was only seen in one GC cell line, KKLS, whilst MKN45 GC cells were still able to metastasize during Wnt5a antibody treatment. Although this evidence does suggests a role for Wnt5a in GC development, further research is needed to elucidate the exact role it has in vivo and where the source of the ligand is coming from (tumor vs. microenvironment) [41]. 

Recently, deregulation of Wnt components at the level of the ligand/receptor has been identified, which in several cancers, including gastric, lung, glioblastoma, breast, melanoma, and prostate, are often more frequent than the deregulation of cytoplasmic components [42]. 

Epigenetic silencing of Wnt antagonists that regulate the pathway at the level of the ligand/receptor have been identified in gastric tumors. Dickkopf 1/2 (DKK1/2) are both antagonists of canonical Wnt signaling that bind to LRP5/6, preventing their interaction with Wnt-Fzd complexes. Wang et al. **[43]** showed that both *DKK1/2* were hyper-methylated in GC patient samples when compared to adjacent normal tissue. Further, hierarchical clustering of GC samples revealed *DKK2* and secreted Frizzled-related protein 2 (*sFRP2*) to be concurrently hypermethylated [43]. sFRPs are a family of five secreted glycoproteins with an extracellular cysteine-rich domain, which downregulate Wnt signaling by binding to Fzd receptors. To corroborate the hyper-methylation of *DKK2* and *sFRP2*, mRNA expression of both genes was analyzed, and showed that gene transcripts were lower in GC samples when compared to normal adjacent tissue, suggesting the hypermethylation was responsible for the silencing of these genes [43]. sFRP-1, 2, or 5 were shown to inhibit Wnt signaling in GC cell lines, which reduced proliferation and increased apoptosis [32] whilst overexpression of sFRP-2 was able to inhibit the proliferation of gastric xenografts [44]. Conversely, sFRP4 is also part of a four-gene single-patient classifier (SPC) signature, reported to help predict which GC patients are at high risk [45], and thus sFRPs have a diverse role in regulating GC biology. 

Adenovirus (chimeric Ad5/35 vector)-mediated *DKK1* overexpression was seen to decrease viability, anchorage-independent colony formation, and invasion of CD44^+^ GC cells through inhibition of canonical Wnt signaling [46]. Further, *DKK1* overexpression significantly impeded the tumorigenesis of CD44^+^ GC cells in vivo [46]. These data suggested that chimeric Ad5/35 vector-mediated *DKK1* overexpression could be a suitable gene therapy for targeting CD44^+^ cancer stem cells in GC. Further, Hong et al. reported that *DKK1* expression was higher in tumors with lymph node metastasis, and patients with high *DKK1* expression had a shorter OS and disease-free survival [47]. 

High expression of the RYK co-receptor (initiates non-canonical Wnt signaling) was found to be correlated with poor differentiation, high TNM stage, and liver metastasis in GC patients (cohort of 250 patients) [48]. Subcutaneous injection of GC cells with stable RYK knockdown into mice showed reduced tumor growth, and increased survival compared to RYK-proficient control mice. Tail vein injection of the RYK knockdown GC cells significantly reduced the number of mice bearing liver tumor nodules (4/12 mice) when compared to control mice (10/12), suggesting RYK plays a role in both GC initiation and metastasis [48]. 

Whole genome sequencing of 100 tumor-normal pairs identified that 54.6% of MSI tumors had mutations in the E3 ubiquitin ligase *RNF43* gene, of which 62.5% were a truncating mutation [5]. Furthermore, a study carried out by Nui et al. [49] demonstrated that mRNA and protein expression of *RNF43* was significantly lower in GC cell lines when compared to normal gastric epithelial cells. This result was corroborated by the same phenotype seen in primary gastric carcinoma tissue when compared to matched normal mucosal tissue [49]. Further, the *RNF43* expression that was seen in the gastric carcinomas was highest in the well-differentiated tumors and lowest in poorly differentiated tumors. A decrease in *RNF43* expression was also seen with the progression of the pTNM stage. Overexpression of *RNF43* in GC cell lines elicited a decrease in proliferation with an increase in apoptotic cells, due to upregulation of p53 and cleaved caspase 3 [49]. 

Functional analysis demonstrated that deletion of *Rnf43* together with closely related *Znrf3* triggered tumorigenesis in the intestine that is dependent on a Wnt-secreting niche. Blocking of this Wnt-secreting niche via porcupine inhibition attenuated the hyperplasia without affecting normal crypts [50]; however, this has yet to be demonstrated in the stomach.

These data demonstrate that deregulation of Wnt signaling at the level of the ligand/receptor can modulate the initiation, growth, and progression of gastric tumors, highlighting that this part of the Wnt pathway is an attractive target for therapeutic intervention in GC. 

To establish whether targeting of the Wnt signaling pathway at the level of the ligand/receptor would be therapeutically beneficial for GC, we recently published a paper identifying that pharmacologically targeting Fzd receptors or specific genetic deletion of *Fzd7* inhibited the initiation and growth of gastric tumors in vitro and in vivo [31]. Here, we review the key findings of that paper and expand on the therapeutic potential of this strategy to target Wnt signaling at the ligand/receptor level. Table 1 summarizes Wnt signaling deregulation in GC. 

## 4. Fzd7 in Gastric Cancer

Of the 10 Fzd receptors, Fzd2, 5, 7, 8, and 9 have been shown to be upregulated in GC tissue [51], with recent evidence from our group that Fzd7 is important in transmitting Wnt signaling in gastric tumors to drive tumor initiation and growth [31]. Overexpression of Fzd7 is seen in late-stage clinical GC, correlating with a significantly shorter survival time, where the median survival time of patients with high Fzd7 expression drops from 77 months to 23.5 months [52]. Further, knockdown of Fzd7 reduced proliferation, migration, epithelial-to-mesenchymal transition (EMT), and expression of stem cell markers in GC cell lines, through inhibition of canonical Wnt signaling [52]. 

Fzd7 is abundantly expressed in GC cell lines, and in the tumors of the Stat3-driven *gp130^F/F^* mouse model of GC [53]. Pharmacologically targeting Fzd receptors with (OMP-18R5/Vantictumab) or inhibiting porcupine with IWP-2 reduced the ability of both *APC* mutant and *APC* wild-type (wt) GC cells from forming anchorage-independent colonies [31]. This emphasizes that Fzd receptors are a therapeutically viable target even in GC tumors with downstream Wnt mutations. Saito-Diaz et al. [54] recently showed that LRP5 knockdown inhibits Wnt signaling in *APC* mutant colorectal cancer cell lines (CRC). However, IWP-2 had no effect on Wnt signaling in the same *APC* mutant CRC cell lines, suggesting that further research is needed to determine which Wnt-driven cancers are sensitive to porcupine inhibitors and what are the molecular mechanisms behind its function. 

The molecular targets of OMP-18R5/Vantictumab are Fzd1, 2, 5, 7, and 8. Both Fzd2/7 were shown to be highly expressed in GC cell lines, thus indicating that either Fzd2/7 was responsible for transmitting Wnt signaling in GC cells [31]. Previous data suggests that Fzd2 is unable to compensate for Fzd7 loss in the intestinal epithelium [55]. Genetically targeting *Fzd7* in vivo or shRNA-targeted knockdown of *Fzd7* in vitro showed that Fzd7 was essential for the growth and initiation of GC cells [31]. 

Deletion of *Fzd7* in the normal gastric epithelium is deleterious, resulting in rapid repopulation with Fzd7-proficient cells [55]. In the gastric adenomas of *Cre^+^ gp130^F/F^ Fzd7^fl/fl^* mice, *Fzd7-*deleted cells were not repopulated but rather survived 20 days post-tamoxifen induction [31] and are thus unable to respond to Wnt signaling, and subsequently failed to proliferate. This mechanism, whereby Fzd7-deficient cells survive in the tumor but do not proliferate, is different from the repopulation observed in the normal gastric epithelium following *Fzd7* deletion and reflects the aberrant biology of tumors compared to the normal epithelium. 

A pivotal risk factor for developing GC is infection with *H. pylori*; however, the mechanism of how infection causes GC is currently unknown. Interestingly, it has been found that *H. pylori* can activate the Wnt/β-catenin pathway through upregulation of Fzd7, which was associated with *H. pylori* infection-induced cell proliferation. Knockdown of *Fzd7* in *H. pylori-*infected GC cells suppressed both cell proliferation and colony formation [56]. To further elucidate how Fzd7 regulates *H. pylori-*infected gastric carcinogenesis, miRNAs involved in GC were explored. Through bioinformatic analysis and functional assays, it was found that miR-27b harbored a putative binding site for Fzd7 3-UTR [56]. Further, it was found that miR-27b was able to suppress *H. pylori* infection and the Wnt signaling pathway through inhibition of Fzd7 [56]. A study carried out by Song et al. **[57]** showed that *H. pylori* caused an upregulation of TRPC6 (transient receptor potential cation channel) expression, by regulating the Wnt/-βcatenin pathway, thus promoting GC progression. Thus, targeting Fzd7 with specific miRNAs could be a therapeutic strategy for GC. 

*c-Myc* is a well characterized β-catenin/TCF target gene within the gastrointestinal tract. Both GC cells and mouse gastric adenomas show upregulation of *c-Myc* in an Fzd7-dependent manner [31]. Conditional deletion of *c-Myc* in *Cre^+^;Apc^fl/fl^;Myc^fl/fl^* mice showed a dramatic reduction of gastric adenoma initiation and Wnt activation compared to the respective controls, identifying *c-Myc* as a key modulator of gastric tumor growth downstream of Fzd7 [31]. Deletion of *c-Myc* in the intestinal epithelium is deleterious and triggers rapid repopulation [58]. We recently identified that this role is not conserved in the gastric epithelium, with the deletion of *c-Myc* leading to no changes in stem cell activity or homeostasis in vivo [59]. Thus, any future therapy in which c-Myc levels are reduced, including targeting Wnt signaling, or specifically *c-Myc* itself for the treatment of GC, would be well tolerated in the stomach.

There are very few functional experiments that decipher the role of other Fzd receptors, besides Fzd7 in GC. Interestingly, Fzd6 is downregulated in GC tissue samples and cell lines, and overexpression of Fzd6 was able to suppress both the proliferation and migration of GC [60], thus Fzd6 would not be a viable therapeutic target for GC. 

## 5. Targeting Wnt Signaling at the Receptor Level in Cells with Downstream Mutations 

Wnt signaling is frequently deregulated in several cancers by mutations of the cytoplasmic components of the pathway, including APC or β-catenin. However, APC mutant cells do not simply switch on Wnt signaling, but rather, regulation is permissible at other levels of the pathway. This is well illustrated by the observation that APC mutant tumors display a variable intensity of nuclear β-catenin, suggesting an environmental factor can regulate APC mutant cells [42,61]. This raises the question of whether Wnt signaling can be targeted at the level of the ligand/receptor even in cells with mutant APC. Recently, Saito-Diaz et al. [54] showed that *APC*^KO^ CRC cells induced the formation of the signalosome (Wnt receptor complex), resulting in activated Wnt signaling. However, CRC cells with *APC* mutations treated with IWP-2 did not inhibit the activation of the signalosome, suggesting that Wnt ligands are dispensable in Wnt pathway activation resulting from *APC* truncation. Interestingly, LRP6 deletion in SW480 and DLD1 cells (both *APC* mutant) did inhibit canonical Wnt signaling and decreased cytoplasmic levels of β-catenin. These data suggest the Wnt receptor signalosome is activated by mutant *APC* and can induce Wnt signaling independent of Wnt ligands. Saito-Diaz et al. [54] also showed that rapid activation of Wnt signaling by the signalosome in *APC* mutant cells was due to internalization of the complex via clathrin-dependent endocytosis. Similarly, recent work from Owen Sansom’s group showed that GTPases RalA and RalB were required for efficient internalization of Fzd7 to activate Wnt signaling in intestinal stem cells [62], illustrating a conserved mechanism of internalization of the signalosome in wt and *APC* mutant cells. 

Schatoff et al. [63] demonstrate that CRC cells with a mutation in the mutation cluster region (MCR) of *APC* can respond to Tankyrase inhibition, suppressing oncogenic signaling in response to AXIN1/2 stabilization. However, CRC cells containing an early truncating mutation (*APC^min^*) were unresponsive to tankyrase inhibition, suggesting repression of Wnt signaling through tankyrase inhibition is highly dependent on specific *APC* disruption. We recently demonstrated that GC organoids derived from *Tff1Cre^ERT2/+;^Apc^fl/fl^* mice treated with the porcupine inhibitor IWP-2, prevented upregulation of the Wnt pathway and was associated with reduced organoid proliferation [31]. These data suggest a difference in how *APC* mutant GC and CRC cells respond to different Wnt inhibitors depending on the location of the mutation in the *APC* gene.

## 6. Wnt Signaling in Metastatic GC 

A recently published study from the Surveillance, Epidemiology and End Results database (SEER) showed that 7792 out of 19,022 (41%) patients presented with metastatic GC, predominantly in the liver (3218 patients) [64]. Another study highlighted that patients undergoing curative gastrectomy had an overall recurrence rate of 21%, and the most common site of metastasis was the peritoneum, followed by liver metastasis, and patients undergoing a curative gastrectomy had para-aortic lymph node (PALN) metastases (8%-28% of patients) [64]. A study carried out by Riihimaki et al. [65] found that male patients with gastric cardia cancer had more metastases found in the nervous system, lung, and bone, whereas patients with non-cardia cancer showed more peritoneal metastases. Patients with metastatic GC have a median survival of 3 months; however, patients with bone or liver metastasis had a worse survival of 2 months [65]. Thus, an understanding the molecular mechanisms driving metastatic disease is essential to develop novel targeted therapies and improve the current abysmal survival rate. 

It is well established that Wnt signaling not only drives the initiation of solid cancers but also contributes to the metastatic progression of the primary tumor. The reactivation of Wnt signaling in the cancer stroma favors cancer stem cell survival. Further, the reactivation of Wnt signaling in the primary tumor aids the epithelial-mesenchymal transition (EMT) of tumor cells, the migration and invasion of tumor cells, and escaping dormancy at metastatic secondary sites [66]. Thus, targeting Wnt signaling is an attractive therapeutic strategy for cancer metastasis. 

Though there are limited studies on Wnt signaling driving metastatic GC, Li et al. [67] discovered that ADAM17 (TNF-α-converting enzyme) mediates GC cell migration through regulation of both the NOTCH and Wnt signaling pathway, evidenced by gene set enrichment analysis. ADAM17 was shown to be highly expressed in primary GC tissue, metastatic lymph node tissue, and in metastatic GC cell lines. Further, knockdown of ADAM17 in a metastatic GC cell line suppressed canonical Wnt signaling through β-catenin downregulation [67]. 

Further, the microRNA miR-544a was found to induce EMT through the activation of Wnt signaling in GC. Specifically, overexpression of miR-544a induced the translocation of β-catenin from the cytoplasm to the nucleus, increasing canonical Wnt signaling in GC cells (MKN28s) [68]. Alongside an upregulation of canonical Wnt signaling, miR-544a upregulation downregulated protein expression of the Wnt destruction complex protein AXIN2 [68].

More so, the stem cell marker LGR5 was found to promote proliferation, invasion, and migration of GC cells through the regulation of canonical Wnt signaling [69] as GC cells treated with the porcupine inhibitor (C-59) dampened LGR5-induced proliferation and migration of GC cells, whereas Wnt3a-treated cells rescued the LGR5-induced phenotype. This was further evidenced by LGR5 overexpression inducing the translocation of β-catenin to the nucleus, and increasing the gene expression of two Wnt gene targets, *AXIN2* and *TCF1* [69].

LGR5 overexpression increased GC cell motility by inducing a morphological change, as cells became elongated with a fibroblast-like appearance and this phenotype was reversed when the GC cells were treated with C-59, suggesting that LGR5 regulates cell migration through Wnt signaling [69]. This is consistent with recent work showing that cytonemes are induced by autocrine Wnt8a binding to the Ror2 receptor [24]. Activation of cytonemes through Wnt8a binding to Ror2 mediates the transport of Wnt8a to surrounding cells, and these receiving cells then trigger canonical Wnt signaling [24], highlighting the cross-talk between non-canonical and canonical Wnt signaling in migrating cells. 

A study carried out by Hanaki et al. [40] discovered that *Wnt5a-*targeted knockdown in GC cell lines reduced cell migration both in vitro and in vivo through inhibition of Rac1 and laminin ϒ2, both key drivers of GC cell invasion. Further, suppression of Wnt5a using an anti-Wnt5a antibody prevented the clathrin-mediated rapid internalization of the Wnt5a-Fzd2 receptor complex [40]. Together, these data identify Wnt signaling either at the ligand/receptor level, or internalization of the receptor complex as an important mechanism driving GC metastasis, and therefore could be considered as attractive therapeutic targets. 

## 7. Clinical Applications of Wnt Inhibitors for GC

Surgery remains the primary modality of cure in GC. Unfortunately, in the UK, approximately 35% of patients present with unresectable or metastatic disease [70]. The surgery offered depends on the location of the tumors. For example, patients with GC in the antrum are offered a subtotal gastrectomy and patients with GC in the body or cardia are offered a total gastrectomy. Despite improvements in the treatment of GC, approximately 25%-30% of patients still develop disease recurrence and ultimately die of their disease, and additional ‘targeted’ treatments are needed [71]. 

**Figure 1:**
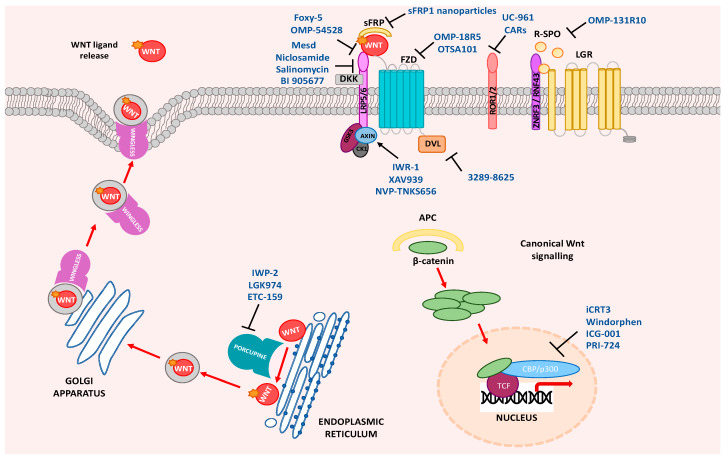
Wnt pathway drug targets. Wnt ligand release, Wnt ligand–receptor interaction, regulators of Wnt antagonists, stabilization of the destruction complex, disheveled (DVL) activation, or disruption of β-catenin co-activators in the nucleus can all be therapeutically targeted. Frizzled (Fzd), R-spondin (R-SPO), leucine-rich repeat containing G protein-coupled receptor (LGR), secreted frizzled-related protein (sFRP), Dickkopf-related protein (DKK), low-density lipoprotein receptor (LRP), adenomatous polyposis coli protein (APC), CREB-binding protein (CBP), casein kinase 1 (CK1), T cell factor (TCF).

It is possible to pharmacologically target Wnt signaling at several places in the pathway (Figure 1), which is portrayed in a number of current clinical trials [13] (Table 2). Table 2 summarizes the current Wnt pathway inhibitors in clinical trials. There are 23 clinical trials that are recruiting or have been completed using the Wnt pathway as a therapeutic target. These trials are exclusively looking at advanced or metastatic disease and these results may not translate to patients undergoing potentially curative surgery, and this is a particularly important future research direction. A precision cancer model using preclinical platforms, such as organoids and tumor genomic sequencing, may facilitate the prescription of adjuvant therapies to patients who have undergone potentially curative treatment who are at a higher risk of relapse. This type of approach is being used in many cancer types where patient-derived organoids are helping to predict patient response to chemotherapy in real time and directly inform clinical decisions [72]. 

Very few of the available pharmacological agents that target the Wnt pathway have been tested in preclinical human GC platforms. This may explain why there are no GC-exclusive Wnt inhibitor trials in the pipeline. Nevertheless, there is evidence to support a program of Wnt inhibitor research in GC. In MKN28 (APC mutant) cells and *Apc* mutant gastric organoids [31], IWP-2, a porcupine inhibitor, suppressed proliferation, and Vanticumab, a Frizzled inhibitor, reduced the number of anchorage-independent colonies [31]. In a phase Ib study of patients with stage IV pancreatic cancer, Ipafricept, an FZD8 inhibitor, in combination with nab-paclitaxel and gemcitabine, resulted in a 34.6% partial response rate and a 46.2% stable disease rate, with 80.8% of patients receiving a clinical benefit [73]. Given that nearly 50% of GC patients have dysregulation of Wnt signaling, these data are promising and worthy of further study. 

Wnt inhibitors are largely very well tolerated in phase 1 clinical trials. The principle adverse event of administering Wnt inhibitors is iatrogenic osteopenia and pathological fractures. Therefore, many therapeutic regimens now incorporate a supplementary bisphosphonate inhibitor, such as zolendronic acid [74]. This has reduced pathological fracture rates from 4.3% [75] to 0% [73]. Other adverse effects include nausea, vomiting, diarrhea, fatigue, and abnormal liver function [75]. Therefore, Wnt inhibitor therapy has a positive pharmacological profile with promising anti-tumor activity and easily managed and reversible adverse event profiles. Interestingly, a recent study showed that ibuprofen can also reduce the proliferation and stemness of GC cells, via inhibition of the Wnt pathway [76]; however, additional in vivo studies will be required to fully understand this process.

## 8. Conclusions

GC represents a heterogenous disease with very variable outcomes, independent of the tumor stage. Deregulation of the Wnt pathway is seen in approximately 50% of tumors [25] and therefore presents a novel therapeutic target. This review demonstrates that, through a higher understanding of the molecular intricacies of the Wnt pathway, several chemotherapeutic agents are currently in clinical trials, targeting the pathway intracellularly and at the receptor/ligand level. Nevertheless, these are primarily recruiting patients with advanced disease who have exhausted other treatment options. Future work will need to strengthen the prognostic evidence for Wnt dysregulation in predicting outcomes in patients with operable disease. This will then strengthen the argument for including Wnt-related adjuvant treatment options in patients at risk of developing recurrence and who have undergone potentially curative treatment, thus reducing recurrence and improving survival. In addition, Wnt signaling also regulates the many molecular processes involved in several stages of metastasis, which is the major cause of the mortality rate in GC patients. Thus, Wnt inhibitors could be effective anti-metastatic drugs for GC. However, considerable changes need to be made to the way anti-metastatic drugs are evaluated in preclinical trials, with a shift in the focus from funding bodies and trial design/patient recruitment to allow this area of therapy to fulfil its huge potential [104]. 

## Figures and Tables

**Table 1:** Summary of Wnt signaling deregulation in GC.

Wnt Component	Role in GC
**Cytoplasmic**	
*APC*	Mutated/deep deletion in GC patient datasets [31]. Promoter hypermethylation in high grade gastric adenomas [33].
β-catenin	Endogenous nuclear expression seen in 13/15 GC cell lines with a subsequent increase in TCF/LEF transcriptional activity [32]. Abnormal nuclear expression seen in high grade gastric adenomas [33].
*AXIN2*	miR-544a targeted protein downregulation in GC cells [68]. 30% of MSI high GCs have a frameshift mutation [36].
*Gsk3*β	Genetic deletion causes rapid gastric tumor formation in mice [37].
**Wnt target genes**	
*MYC*	Gene amplification in GC patient samples [36]. GC cells and mouse adenoma show gene upregulation in an Fzd7-dependent manner [31].
LGR5	Overexpression regulates GC cell proliferation, migration, and invasion [69].
**Wnt ligands**	
*WNT1*	Upregulated in human GC tissue. Overexpression accelerates gastric cancer stem cells [26].
*WNT2b*	Upregulated in GC tissue [27].
*Wnt3a*	Upregulated in gp130F/F gastric tumors [31].
WNT5a	High protein expression in GC patient samples, positively associated with the depth of tumor invasion and degree of lymph node metastasis [28].
*WNT6*	Upregulated in GC patient samples and GC cell lines. Expression positively correlated with tumor stage and node status [29].
*WNT10a*	Upregulated in GC cells and primary GC tissue [30].
**Wnt antagonists**	
*DKK1*	Hypermethylated in GC patient samples [46].
*DKK2*	Hypermethylated in GC patient samples + gene transcripts lower in GC patient samples [43].
*sFRP2*	Concurrently hypermethylated with *DKK2* + gene transcripts lower in GC patient samples [43].
**Wnt receptors**	
RYK co-receptor	High expression correlated with poor differentiation, high TNM stage and liver metastasis in GC patients [48].
*RNF43*	Truncating mutation in MSI GC tumors [5]. Protein expression is significantly lower in GC cells than normal gastric epithelial cells [49].
*FZD2*	Upregulated in GC cells (TMK1, MKN7, MKN28, MKN45, MKN74, and KATO-III) and in 4/10 primary GC tissue [51].
*FZD5*	Upregulated in GC cells (MKN45) [51].
*FZD7*	Overexpression is seen in late-stage clinical GC, correlating with a decrease in survival time [52]. Knockdown significantly reduces GC cell proliferation, migration, EMT, and expression of stem cell markers [52].
*FZD8*	Upregulated in 4/10 primary GC tissue [51].
*FZD9*	Upregulated in 2/10 primary GC tissue [51].

**Table 2:** Wnt pathway inhibitors in current clinical trials.

WNT PATHWAY TARGET	DRUG	PHASE AND CLINICAL TRIAL		CANCER TYPE
**PORCUPINE**	LGK974 (WNT974)	Phase I NCT01351103 Phase I/II NCT02278133	[77]	Pancreatic Cancer, BRAF mutant CRC, Melanoma, Triple negative Breast Cancer, Head and Neck Squamous Cell Cancer, Cervical Squamous Cell Cancer, Esophageal Squamous Cell Cancer, Lung Squamous Cell Cancer Metastatic Colorectal cancer
	ETC-1922159	Phase IA/B NCT02521844	[78,79]	Advanced or metastatic solid tumors
**RSPO3**	OMP131R10	Phase I NCT02482441	[80]	Metastatic Colorectal Cancer, advanced relapsed or refractory solid tumors
**WNT5A RECEPTOR**	Foxy-5	Phase I NCT02020291	[81]	Metastatic Breast, Colon or Prostate cancer (loss or reduced Wnt5a on IHC)
		Phase I NCT02655952	[82]	Metastatic Breast, Colon or Prostate cancer (loss or reduced Wnt5a on IHC)
**WNT FAMILY**	OMP-54F28 (Ipafricept)	Phase I NCT01608867	[83]	Metastatic or unresectable solid tumors
		Phase I NCT02092363	[84]	Ovarian, primary peritoneal or fallopian tube cancer
		Phase I NCT02069145	[85,86]	Locally advanced or metastatic Hepatocellular Carcinoma
		Phase I NCT02050178	[87,88]	TNM stage IV Ductal adenocarcinoma of the pancreas
**FZD1,2,5,7,8**	OMP-18R5 (Vantictumab)	Phase I NCT01345201	[89]	Metastatic solid tumors with no other standard treatment options
		Phase I NCT01957007	[90]	Recurrent of TNM stage IV Non-small cell lung cancer
		Phase I NCT01973309	[91]	Recurrent or metastatic breast cancer (HER2 overexpression not eligible)
		Phase I NCT02005315	[92]	TNM stage IV Ductal adenocarcinoma of the pancreas
**FZD10**	OTSA101	Phase I NCT01469975	[93,94]	Progressive synovial sarcoma
**ROR1**	UC-961 (Cirmtuzumab)	Phase I NCT02222688	[95]	Relapsed or refractory B cell Chronic Lymphocytic Leukemia (CLL)
		Phase I NCT02860676	[96]	Relapsed or refractory B cell CLL
		Phase I/II NCT03088878	[97]	B Cell CLL, Small Cell Lymphocytic Lymphoma, Mantle Cell Lymphoma
		Phase I NCT02776917	[98]	Metastatic or locally advanced HER2 negative breast cancer
**CREB BINDING PROTEIN**	PRI-724	Phase I NCT01302405	[99]	Metastatic or unresectable solid tumors
		Phase I NCT01764477	[100]	Relapsed, locally advanced or metastatic pancreatic adenocarcinoma
		Phase I/II NCT01606579	[101,102]	Relapse or refractory Acute Myeloid Leukemia, advanced Chronic Myeloid Leukemia
**LRP5/6**	BI 905677	Phase 1 NCT03604445	[103]	Metastatic or unresectable solid tumors

## Abbreviations

APC	Adenomatous polyposis coli
β-TrCP	Beta-Transducin Repeat Containing E3 Ubiquitin Protein Ligase
CAMKII	calmodulin dependent protein kinase II
CBP	CREB-binding protein
CDC	Cell Division Cycle 42
CDK	Cyclin Dependent Kinase
CK1α	Casein kinase 1α
CREB	CAMP Responsive Element Binding Protein
DAAM	Dishevelled Associated Activator Of Morphogenesis
DAG	1,2-diacylglycerol
DGO	Diego
DKK	Dickkopf
DVL	Dishevelled
FZD	Frizzled
FMI	Flamingo
GC	gastric cancer
GSK3α/β	Glycogen synthase 3
IP3	Inositol 1,4,5-triphosphate
JUN	Jun Proto-Oncogene, AP-1 Transcription Factor Subunit
LGR	Leucine-rich repeat-containing G-protein-coupled receptor
LRP	Low-density lipoprotein receptor-related protein
NFκB	Nuclear Factor Kappa B Subunit 1
OC	Oesophageal cancer
PK	LIM domain protein Prickle
PLC	Phospholipase C
pTNM stage	Pathological Tumor-Node-Metastasis stage
RAC	Rac Family Small GTPase
RHO	Rhodopsin
ROCK	Rho Associated Coiled-Coil Containing Protein Kinase
ROR	Receptor Tyrosine Kinase Like Orphan Receptor
RNF	Ring finger
Ryk	Receptor Like Tyrosine Kinase
R	SPO—Rspondin
SFRP	Secreted Frizzled-related protein
SDC	Syndican4
TAZ	Taffazin
TCF/LEF	T-cell factor/lymphoid enhancer factor
TLE	Groucho/ transducin-like Enhancer of Split
VANG	Van Gogh transmembrane protein
YAP	Yes associated protein
VANG	Van Gogh transmembrane protein
YAP	Yes associated protein
